# Genomic analysis identifies frequent deletions of *Dystrophin* in olfactory neuroblastoma

**DOI:** 10.1038/s41467-018-07578-z

**Published:** 2018-12-21

**Authors:** Gary L. Gallia, Ming Zhang, Yi Ning, Michael C. Haffner, Denise Batista, Zev A. Binder, Justin A. Bishop, Christine L. Hann, Ralph H. Hruban, Masaru Ishii, Alison P. Klein, Douglas D. Reh, Lisa M. Rooper, Vafi Salmasi, Rafael J. Tamargo, Qing Wang, Tara Williamson, Tianna Zhao, Ying Zou, Alan K. Meeker, Nishant Agrawal, Bert Vogelstein, Kenneth W. Kinzler, Nickolas Papadopoulos, Chetan Bettegowda

**Affiliations:** 1 Department of Neurosurgery, Johns Hopkins University School of Medicine, Baltimore, MD 21287 USA; 2 Department of Otolaryngology – Head and Neck Surgery, Johns Hopkins University School of Medicine, Baltimore, MD 21287 USA; 3 Department of Oncology, Johns Hopkins University School of Medicine, Baltimore, MD 21287 USA; 4 Ludwig Center for Cancer Genetics and Therapeutics, Department of Oncology and Pathology and the Sidney Kimmel Comprehensive Cancer Center, Johns Hopkins University School of Medicine, Baltimore, MD 21287 USA; 5 Department of Pathology, Johns Hopkins University School of Medicine, Baltimore, MD 21287 USA; 6 Department of Pathology, University of Maryland School of Medicine, Baltimore, MD 21201 USA; 7 Howard Hughes Medical Institutes, Johns Hopkins University School of Medicine, Baltimore, MD 21287 USA; 8 Department of Radiation Oncology, Johns Hopkins University School of Medicine, Baltimore, MD 21287 USA; 9 Present Address: Department of Neurosurgery, University of Pennsylvania, Philadelphia, PA 19104 USA; 10 Present Address: Department of Pathology, University of Texas Southwestern Medical Center, Dallas, TX 75390 USA; 11 Present Address: Department of Anesthesiology, Perioperative and Pain Medicine, Stanford University School of Medicine, Palo Alto, CA 94305 USA; 12 Present Address: Department of Surgery, Division of Otolaryngology and Head and Neck Surgery, University of Chicago, Chicago, IL 60637 USA

## Abstract

Olfactory neuroblastoma (ONB) is a rare malignant neoplasm arising in the upper portion of the sinonasal cavity. To better understand the genetic bases for ONB, here we perform whole exome and whole genome sequencing as well as single nucleotide polymorphism array analyses in a series of ONB patient samples. Deletions involving the dystrophin (*DMD*) locus are found in 12 of 14 (86%) tumors. Interestingly, one of the remaining tumors has a deletion in *LAMA2*, bringing the number of ONBs with deletions of genes involved in the development of muscular dystrophies to 13 or 93%. This high prevalence implicates an unexpected functional role for genes causing hereditary muscular dystrophies in ONB.

## Abstract (web summary)

Olfactory neuroblastoma (ONB) is a rare malignant tumor whose genetic basis is poorly understood. Here the authors identify recurrent deletions involving dystrophin in the majority of ONB patient tumors examined.

## Introduction

Olfactory neuroblastoma (ONB), also known as esthesioneuroblastoma (ENB), is a rare malignant neoplasm of the sinonasal cavity first described in 1924^1^. It has an incidence of 0.4 cases per million and accounts for ~6% of all sinonasal malignancies^2,3^. ONB is thought to arise in the olfactory epithelium at the anterior skull base and superior aspect of the sinonasal cavity^4^. These locally aggressive neoplasms invade the nasal cavity, paranasal sinuses, cribriform plate, intracranial space, and orbit and can metastasize to the neck, central nervous system, and bone^4,5^. In cases amenable to surgery, resection followed by postoperative radiotherapy is the most commonly used treatment; for patients with unresectable or metastatic disease, chemotherapy and radiation are used^4^. Despite multi-modality therapy, the 10-year overall survival rate for patients with ONB is ~50%^4,6^. Therefore, there is a great need for a better understanding of the pathogenesis of this tumor type, which could inform improved diagnostic and therapeutic strategies.

There are limited data on the biological basis and genomic constitution of ONB and, to date, no recurrent genetic alterations have been identified^7–14^. To examine the genomic landscape of ONB, here we perform whole-exome sequencing (WES), whole-genome sequencing (WGS), and single nucleotide polymorphism (SNP) array analyses on a series of clinically well-characterized ONB patient samples. These studies demonstrate that ONB has frequent somatic deletions involving the *DMD* locus and implicates a central role for genes causing hereditary muscular dystrophies in ONB pathogenesis.

## Results

### Genomic characterization of ONB by WES and WGS

Initially, DNA from 11 matched ONB tumor and normal samples (nine male and two female; Supplementary Table 1) underwent WES. The average number of reads per targeted base was 140.0 ± 23.1 for tumor samples and 157.6 ± 18.9 for matched normal DNA derived from peripheral blood, with over 90% of targeted bases having at least 10 distinct reads (Supplementary Table 2). On average, each tumor harbored 13.2 ± 5.6 (range 4–21) somatic mutations. The only recurrent intragenic mutation seen in this cohort was in *TTN*, which was observed in two cases (ENB01PT and ENB05PT) (Supplementary Data 1). Given the lack of shared genetic drivers discovered via WES, we reasoned that other genetic alterations, like re-arrangements, that cannot be readily detected by WES could be present in these tumors. To determine this, we performed WGS on 6 of the 11 cases. The average number of reads per targeted base with WGS was 29.0 ± 5.3 for the six tumor specimens and 35.0 ± 4.6 for the matched normal samples, with over 93% of bases represented by at least 10 reads (Supplementary Table 3). We identified large deletions involving the X chromosome in five of these six samples. In each case, the deletion spanned the *DMD* locus (Supplementary Figure 1), which prompted further evaluation of this region.

### SNP array analyses confirm recurrent loss of Xp21.1

**Fig. 1:**
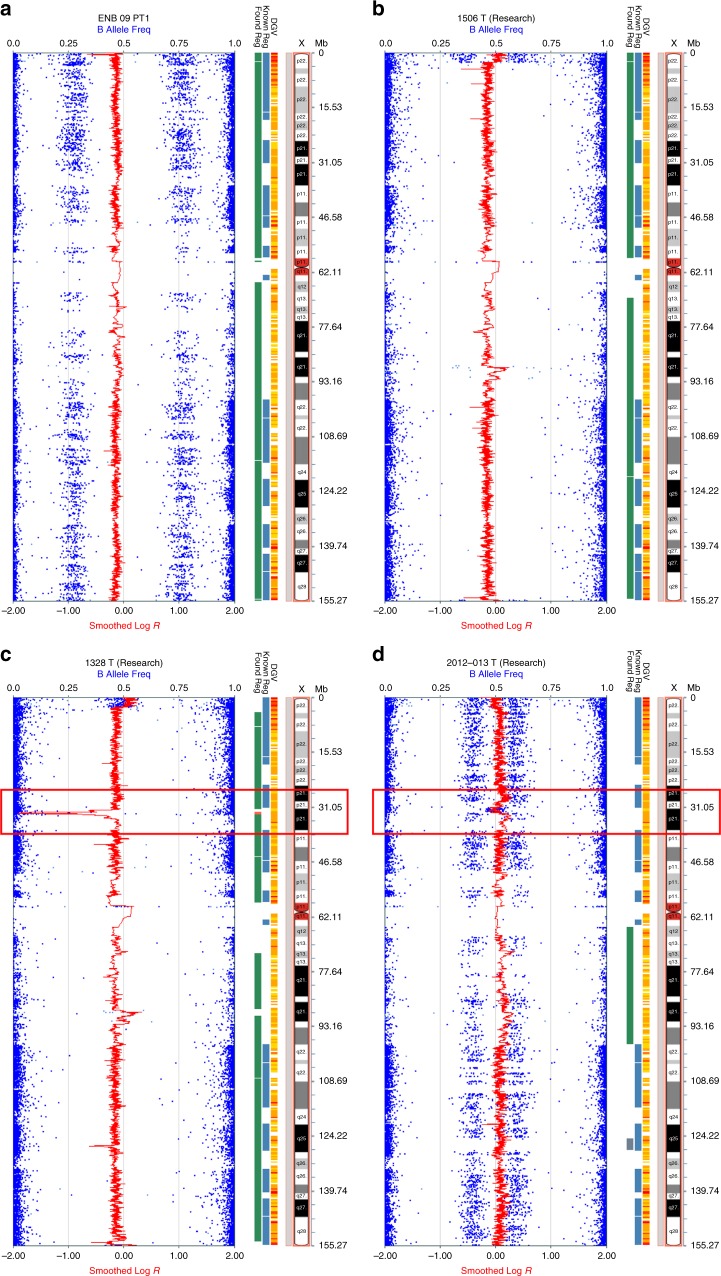
SNP array analysis of X chromosomes in representative ONB tumor samples. **a** An ONB, from a female patient, with mosaic loss of the X chromosome (ENB09PT1, Table 1) as indicated by the left shift of Log *R* and change of B allele frequencies. **b** An ONB, from a male patient, with no evidence of deletion in *DMD* (ENB1506T, Table 1). **c** An ONB, from a male patient, harboring a deletion in *DMD* (ENB1328T, Table 1). **d** An ONB, from a female patient, harboring a deletion in *DMD* (ENB2012-13T, Table 1); the right shift of Log *R* and change of B allele frequencies indicate the presence of mosaicism with relative gain of the X chromosome and deletion in *DMD* in a percentage of the tumor cells

To investigate global genomic losses or gains that are common in ONB, we next used a SNP array approach. Initially, SNP array studies with an Illumina Beadchip containing 300,000 markers were performed on 10 ONB tumor samples and multiple abnormalities were observed. Most abnormalities involved copy number changes (loss or gain) of entire copies of chromosomes. Given the extent of copy number alterations, it was not possible to precisely determine tumor cell ploidy status based on the SNP array. Therefore, we focused our analysis on structural changes, such as deletions and duplications, within chromosomes. Similar to the WGS data, we observed recurrent deletions in chromosome Xp21.1. Deletions were observed in 9 of the 10 tumors (Table 1). In seven of the eight tumors from male patients, these deletions involved the *DMD* coding region and in five of these seven tumors, the deletion extended into the 5′ UTR. In the tumor from an additional male patient (ENB7PT2), the deletion was 102 kb upstream to the *DMD* gene but encompassed three CTCF-binding motifs that could affect transcriptional activity. One tumor from a female patient (ENB09PT1) showed loss of one allele of chromosome X (Fig. 1a). Only one of the 10 tumors (from the other female patient, ENB03PT2) did not show any detectable structural abnormality in the X chromosomes. Interestingly, this patient did have a homozygous deletion in *LAMA2*, the gene encoding the alpha 2 chain of laminin (Supplementary Figure 2).

**Table 1:** SNP array evaluation of chromosome X in ONB

Sample #	Specimen name	Sex	Sample type	Chromosome X abnormalities	Deletion size (kb)	*DMD* exons deleted
1	ENB01PT	M	Tumor	Deletion in Xp21.1: 32,781,748–33,488,489	707	1–7
2	ENB03PT2	F	Tumor	No deletion in Xp21.1		
3	ENB04PT	M	Tumor	Deletion in Xp21.1: 32,999,013–33,712,422	713	1–2
4	ENB05PT	M	Tumor	Deletion in Xp21.1: 32,917,250–33,712,422 and 33,907,073–34,070,287	795 163	1–2
5	ENB7PT2	M	Tumor	Deletion in Xp21.1: 33,459,497–33,712,422	253	^a^
6	ENB08PT2	M	Tumor	Deletion in Xp21.1: 32,615,422–33,273,667	658	2–12
7	ENB09PT1	F	Tumor	Relative loss of X (Fig. 1a)		
8	ENB10PT	M	Tumor	Deletion in Xp21.1: 32,244,312–32,481,863 and 32,775,043–33,136,164	238 361	25–43 2–7
9	ENB12PT	M	Tumor	Deletion in Xp21.1: 33,199,451–34,021,156	822	1
10	ENB606PT2	M	Tumor	Deletion in Xp21.1: 32,405,747–33,531,257	1126	1–32
11T	ENB1328T	M	Tumor	Deletion in Xp21.1: 32,563,263–33,314,650 (Fig. 1c)	751	2–17
11B	ENB1328B	M	Blood	No reportable abnormality in genome		
12T	ENB1506T	M	Tumor	No deletion in Xp21.1 (Fig. 1b)		
12B	ENB1506B	M	Blood	No reportable abnormality in genome		
13T	ENB2012–013T	F	Tumor	Relative gain of X and a deletion in Xp21.1: 30,323,916–32,772,054 (Fig. 1d)	2448	8–79
13B	ENB2012–013B	F	Blood	No reportable abnormality in genome		
14T	ENBBG-T	M	Tumor	Deletion in Xp21.1: 32,814,846–32,842,232 (Fig. 2)	27	5–7
14B	ENBBG-B	M	Blood	No reportable abnormality in genome		

All the deletions are in Xp21.1, with genomic positions indicated according to the Human Genome Build 37 (hg19). The *DMD* gene (NM_000109) is at chrX: 31,137,345–33,357,726

^a^Sample #5 has a deletion involving the 5′UTR which is predicted to affect CTCF-binding domains

**Fig. 2:**
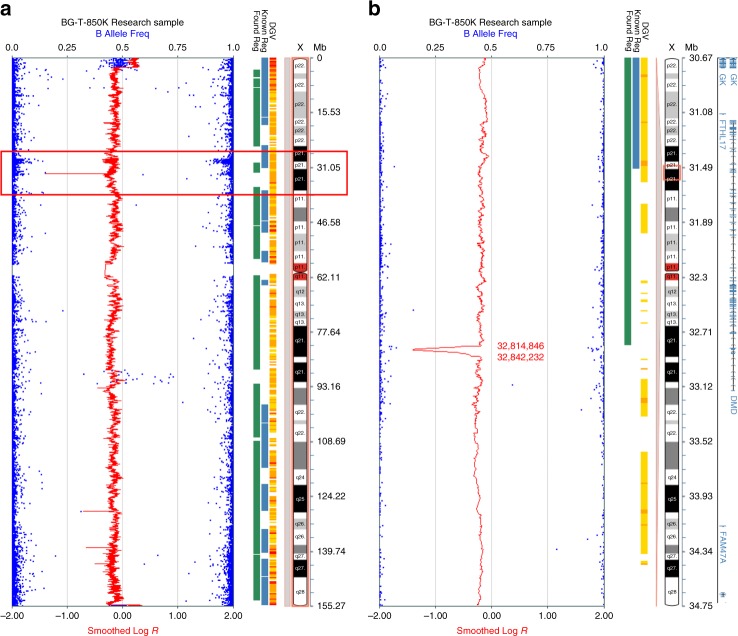
Detection of a 27 kb deletion in *DMD*. High density SNP array (850k chip) detected a 27 kb deletion of the *DMD* gene in an ONB from a male patient (ENBBG-T, Table 1). The panel on the left (**a**) shows the array profile of the entire chromosome X, with indicated deletion in Xp21.1. The panel on the right (**b**) focuses on the region of Xp21.1 containing the *DMD* gene. The deletion is within the *DMD* gene, from genomic position 32,814,846–32,842,232 (hg19)

Intrigued by these findings, we extended our SNP array analyses by using the Affymetrix SNP 6.0 chip to investigate ONB tumor samples and normal tissue from an additional four patients (samples 11T–14B, Table 1, Supplementary Table 1). Deletions in Xp21.1 were detected in tumor samples from two male patients (ENB1328T, Fig. 1c and ENBBG-T, Fig. 2) and from one female patient (ENB2012-013T, Fig. 1d); one male patient did not show a deletion in *DMD* (ENB1506T, Fig. 1b). The female patient showed evidence of relative gain of chromosome X and also a deletion in Xp21.1. All three deletions spanned the *DMD* locus. Two of the *DMD* deletions were large (751 kb and 2.4 Mb) and the other (in sample ENBBG-T) was small. We applied a high density Illumina Beadchip containing 850,000 markers to further verify the small deletion in ENBBG-T and confirmed the presence of a 27 kb deletion within the *DMD* gene (Fig. 2). In all cases harboring deletions, evaluation of DNA from the matched peripheral blood, using the same analytic methods, showed that the deletions were somatic rather than germline. Taken together, abnormalities of Xp21.1 involving the *DMD* locus were found in 12 of 14 tumors (86%) (Supplementary Figure 3).

### Verification of *DMD* deletion status in tissue specimens

To verify the *DMD* deletions by an additional orthogonal in situ approach, we generated custom fluorescent in situ hybridization (FISH) probes covering a deletion interval common to several tumors containing *DMD* deletions (Supplementary Figure 4). Tissue was of sufficient quality to obtain FISH signals in four tumors. Two cases with deletions involving *DMD* and one case with an intact *DMD* locus were validated using FISH (Supplementary Figure 4). The fourth case tested (ENB7PT2) had a deletion outside of the region targeted by the FISH probe. This case showed maintained FISH signals, further demonstrating the specificity of the assay.

### Examination of non-ONB tumors for *DMD* deletions

Next, in order to demonstrate that the deletions in the *DMD* locus were not stochastic, we analyzed WGS data from 12 additional non-ONB tumors that were sequenced in a similar fashion to the ONB samples. This cohort of tumors included five malignant peripheral nerve sheath tumors, four gastric cancers, and three pancreatic neuroendocrine tumors. None of these 12 non-ONB tumors harbored alterations in the *DMD* locus and, when compared to the prevalence of deletions in ONBs, this difference was statistically significant (*p* < 0.01; Fisher’s Exact Test). We next interrogated the COSMIC database which lists copy number analysis on 1093 central nervous system neoplasms, 34 (~3%) of which were reported to have a deletion involving *DMD*^15^. Upon closer inspection, each case involved a large segment chromosomal loss and none demonstrated focal deletions involving the *DMD* locus.

## Discussion

*DMD* is the largest known human gene, spanning greater than 2 Mb, in the genome and encodes the protein dystrophin^16^. By interacting with other proteins including dystroglycan, sarcoglycan, dysferlin, and others in cardiac and skeletal muscle, dystrophin forms a bridge across the sarcolemma connecting the cytoskeleton with the extracellular matrix^16^. Genomic alterations of *DMD* are well-known causes of dystrophinopathies including Duchene muscular dystrophy (DMD), Becker muscular dystrophy (BMD), and X-linked dilated cardiomyopathy (XLDC)^16^. In this study, we demonstrated somatic alterations in the *DMD* locus occurring in 12 of 14 ONBs.

One of the two tumors in this study with no change in Xp21.1 had a somatic homozygous deletion in *LAMA2* and, interestingly, mutations in this gene have been identified as the cause of merosin-deficient congenital muscular dystrophy^17^. *LAMA2* has also been implicated in a number of other cancers and mutations in this gene have been identified in 14% of hepatocellular carcinomas and aberrant methylation in 80% of colorectal cancers^18,19^. In sum, 13 of our 14 ONBs had a somatic deletion in the genes that are involved in muscular dystrophies, perhaps providing new insights into the pathophysiology of these tumors.

In addition, two tumors had non-synonymous point mutations in *TTN*, a gene that encodes titin, a protein central to muscle structure and function. Mutations in this gene have been reported in numerous cardiac and skeletal muscle disorders including limb-girdle muscular dystrophy type 2J and tibial muscular dystrophy^20^. However, both cases with mutations in the *TTN* gene also had deletions in *DMD* suggesting that it is not a driver of ONB pathogenesis. In addition, unlike *DMD* and *LAMA2* which were deleted, only missense mutations were seen in *TTN*, as has been reported in a number of other tumor types and is not unexpected given the large size of the *TTN* gene^21,22^.

Interestingly, mice lacking dystrophin or dystrophin-associated proteins including dysferlin, α sarcoglycan, calpain-3, and Large form spontaneous malignant tumors with muscle differentiation^23–26^. *DMD* deletions have also been identified in human malignancies. *DMD* deletions were reported in 3 of 55 (5.5%) melanoma cell lines^27^. More recently, *DMD* deletions were demonstrated in 25 of 40 (63%) high-grade myogenic cancers using SNP analysis, including 19 of 29 (66%) gastrointestinal stromal tumors, 3 of 4 (75%) rhabdomyosarcomas (RMS), and 3 of 7 (43%) leiomyosarcomas^28^. In contrast, *DMD* deletions were not seen in 58 non-myogenic sarcomas and were only reported in 4.3% of non-sarcoma human cancer cell lines in the Cancer Cell Line Encyclopedia^28^. Another recent study found *DMD* alterations in 3.4% of 8052 samples in the cBioPortal database, although interestingly, *DMD* alterations were not seen in RMS^29^. In our study, we found somatic alterations that affect the *DMD* locus in 86% of ONBs. Although loss of the entire X chromosome, as well as Xp21.1 have been reported in prior cytogenetic studies of ONB^9,13^, our report demonstrates that these deletions target *DMD* and that they occur in a very high fraction of these malignancies.

The *DMD* gene encodes various dystrophin isoforms^16^. In our ONB cohort, *DMD* deletions were concentrated in the 5′ end of the gene, with eight tumors harboring deletions eliminating exons 1 and/or 2. An additional tumor had a deletion involving exons 5–7, another involving exons 8–79, and a third that deleted regulatory elements in the 5′ UTR. We predict this mutation profile would affect transcription of the full-length 427-kDa isoform, while preserving transcription of smaller isoforms, such as p71 (encoded by exons 63–79) in most tumors. Similarly, in the study by Wang et al. using a multiplex ligation-dependent probe amplification assay, deletions were found in 24 of 56 (43%) high-grade myogenic cancers, all of which were predicted to abrogate transcription of full length DMD and p71 was found to be preserved in cancers with *DMD* deletion^28^.

From a functional standpoint, dystrophin is best known for its structural role in skeletal and cardiac muscle cells^16^, but there are burgeoning data, from both melanocytic and myogenic model systems, that dystrophin functions as a tumor suppressor^27,28^. The high rate of *DMD* deletions found in ONBs coupled with the emerging tumor suppressor activity of dystrophin suggests a central role for *DMD* in the pathogenesis of this tumor type.

## Methods

### Tumor and matched normal sample preparation

Fresh-frozen tumor samples and matched blood were obtained from patients under Intuitional Review Board-approved protocols at the Johns Hopkins Hospital after informed consent. A board certified head and neck pathologist verified the diagnosis. Tumor tissue was macrodissected to remove normal tissue and enhance neoplastic content, as confirmed by serial frozen sections. All tumor specimens utilized for analysis had an estimated neoplastic content >70%.

### Whole-exome and WGS

Genomic DNA libraries were prepared and captured following Illumina’s suggested protocol. The SureSelectXT Human All Exon V6 kit was used to capture the coding sequences from individual libraries for each sample (Agilent). The libraries were sequenced using the Illumina HiSeq Genome Analyzer, yielding 100 base pairs of sequencing information that was used for whole-genome and whole-exome analyses^30,31^. Following the completion of sequencing, the data were retrieved and analyzed in silico to determine overall coverage and read quality. Sequencing reads were analyzed and aligned to human genome hg18 with the Eland algorithm in CASAVA 1.7 software (Illumina). All low-quality, poorly aligned, or dbSNP-containing reads were removed from further analysis. Reads were mapped using the default seed-and-extend algorithm, which allowed a maximum of two mismatched bases in the first 32 bp of sequence. Identification of somatic alterations was performed as previously described^32^. Briefly, a mismatched based was identified as a mutation after meeting the following criteria: (i) it was present in more than five distinct reads; (ii) the number of distinct reads containing the mismatched base was at least 10% of the total distinct reads; and (iii) it was present in <0.5% of the reads in the matched normal sample. “Distinct reads” were defined as fragments that had different sequences at either the 5′ or 3′ end, thereby indicating that they originated from different template molecules.

### SNP array studies

SNP array analysis was performed using Illumina Human CytoSNP12 Beadchip containing over 300,000 markers. Allele ratios and signal intensity were analyzed with the copy number variation (CNV) Partition 2.4.4.0 algorithm in KaryoStudio (v.1.4.3.0) and GenomeStudio (v.2010.3) (Illumina). SNP array studies were repeated on Affymetrix SNP 6.0 chip for 4 tumor samples, in parallel with their matched peripheral blood samples. The raw Affymetrix CEL files were converted to allele-specific signals and genotype calls using Affymetrix Power Tools and BirdSeed algorithm to calculate log *R* ratio and B allele frequency values. The raw data were analyzed with the Genotyping Console software 4.2 (Affymetrix). One sample with <30 kb deletion was further verified using Illumina Human Infinium CytoSNP-850K v1.1 Beadchip containing over 850,000 markers. Genomic positions are based on the Human Genome Build 37 (hg19).

### *DMD* locus-specific and X centromere FISH

Custom fluorescein-labeled FISH probe made from BAC RPCI-11 clone 98K7 was ordered from Empire Genomics. This BAC contains sequences within the *DMD* locus on the X chromosome covering a deletion interval common to several of the cases identified in this study. Spectrum Orange labeled probe specific for the X centromere was obtained from Abbott Molecular. Tissue pre-treatment was conducted as follows: 5 µm sections were cut to charged slides from FFPE tissue blocks, deparafinized in xylenes, rehydrated through graded alcohols, treated with 0.2 N HCl for 15 min at room temperature, immersed in citrate buffer at 80 °C, and incubated for 40 min. Following heat treatment, slides were immersed in 2X SSC, then in distilled water followed by application of 0.2 N HCl at room temperature for 2 min. Slides then underwent protease pretreatment on an automated slide handling platform (Roche/Ventana). Digestion was set between 24 and 28 min, depending on assessment of optimal digestion time uniquely determined for each case. Following digestion, slides were rinsed in water, immersed in 10% neutral buffered formalin for 5 min at room temperature, washed with water, dehydrated through increasing alcohols and air dried. Hybridization was conducted as follows: pre-treated slides were heated to 45 °C on a Thermobrite slide hybridization platform (Thermofisher Scientific). *DMD* locus probe and X centromere probe were applied in hybridization buffer (Empire Genomics) to the tissue, cover slip was applied and sealed with rubber cement. Co-denaturation was conducted by increasing temperature to 95 °C for 5 min, followed by hybridization at 37 °C for 20 h. Cover slips were removed and slides washed in 2X SSC/0.3% Igepal for exactly 2 min at 72 °C, then transferred to a 2x SSC/0.1% Igepal for 60 s at room temperature. Slides were then air dried, counterstained with DAPI, and washed in water. Anti-fade solution (Prolong Gold, Thermofisher Scientific) was applied, followed by cover slipping. Slides were visualized by fluorescence microscopy using a Nikon 50i epifluorescence microscope and monochrome greyscale images captured using a ×100 oil immersion objective and digital camera (Photometrics). Captured images were pseudocolored and merged in Nikon Elements software. Probes were validated by hybridizing onto control normal donor metaphase slides (Abbott Vysis) and confirming expected localization to the X chromosome.

## Electronic supplementary material

Supplementary Information

Supplementary Data 1

Description of Additional Supplementary Files

## Data Availability

Whole exome and genome sequencing data generated during this study are available in the European Genome-Phenome Archive (http://www.ebi.ac.uk/ega; accession number EGAS00001003225).
