# Supplementary material for: Genomic analysis identifies frequent deletions of Dystrophin in olfactory neuroblastoma
Source: Nat Commun. 2018 Dec 21;9:5410. doi: 10.1038/s41467-018-07578-z (PMC6303314; doi:10.1038/s41467-018-07578-z)
Supplement: Supplementary file 1 — Supplementary Information [file 41467_2018_7578_MOESM1_ESM.pdf]

Genomic Analysis Identifies Frequent Deletions of *Dystrophin* in Olfactory Neuroblastoma

Gallia et al.

Supplementary Information

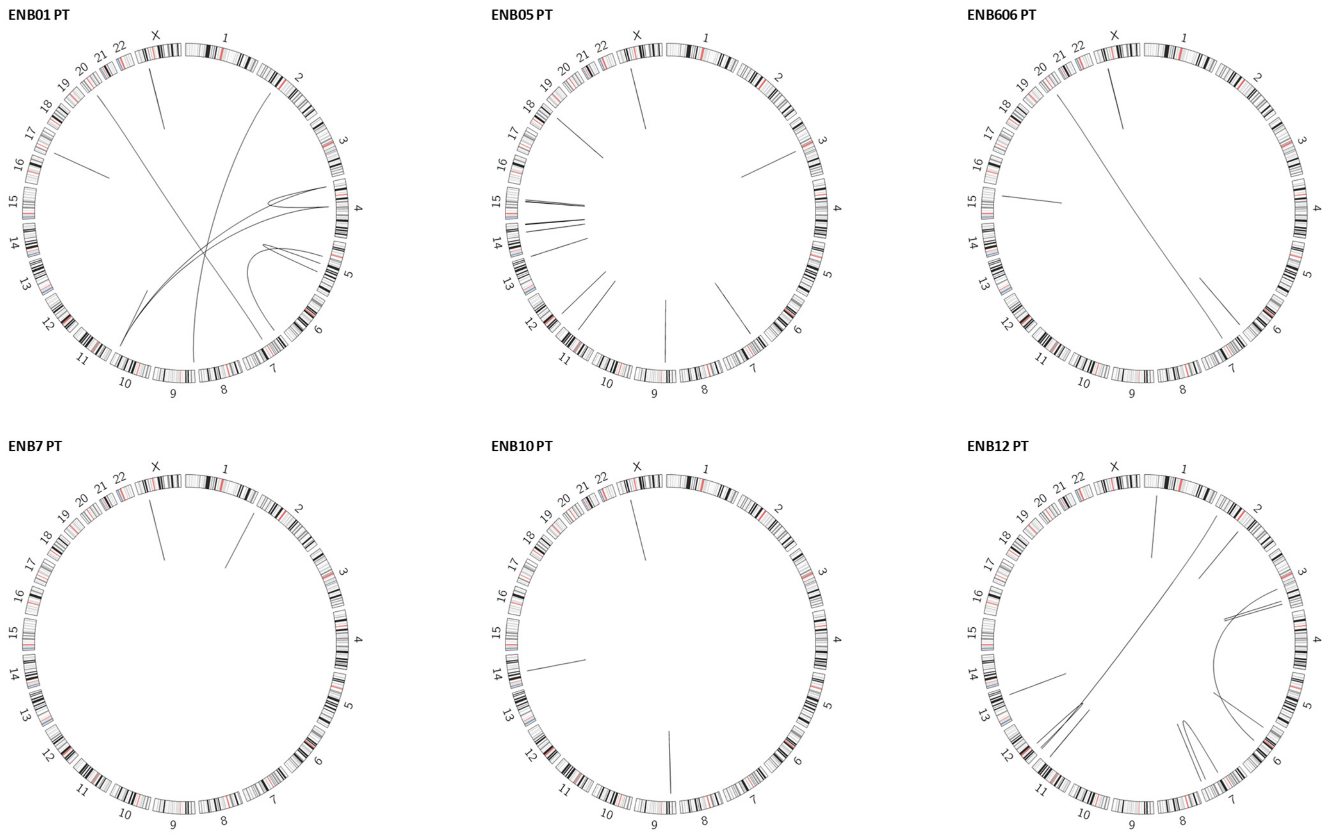

Supplementary Figure 1. Circos plots demonstrating chromosomal aberrations as determined via WGS. ONB tumors demonstrated a range of structural alterations from 2-13 per tumor. Deletions involving the *DMD* locus on the X chromosome were the only recurrent change, found in 5 of 6 patients (all except ENB12PT).

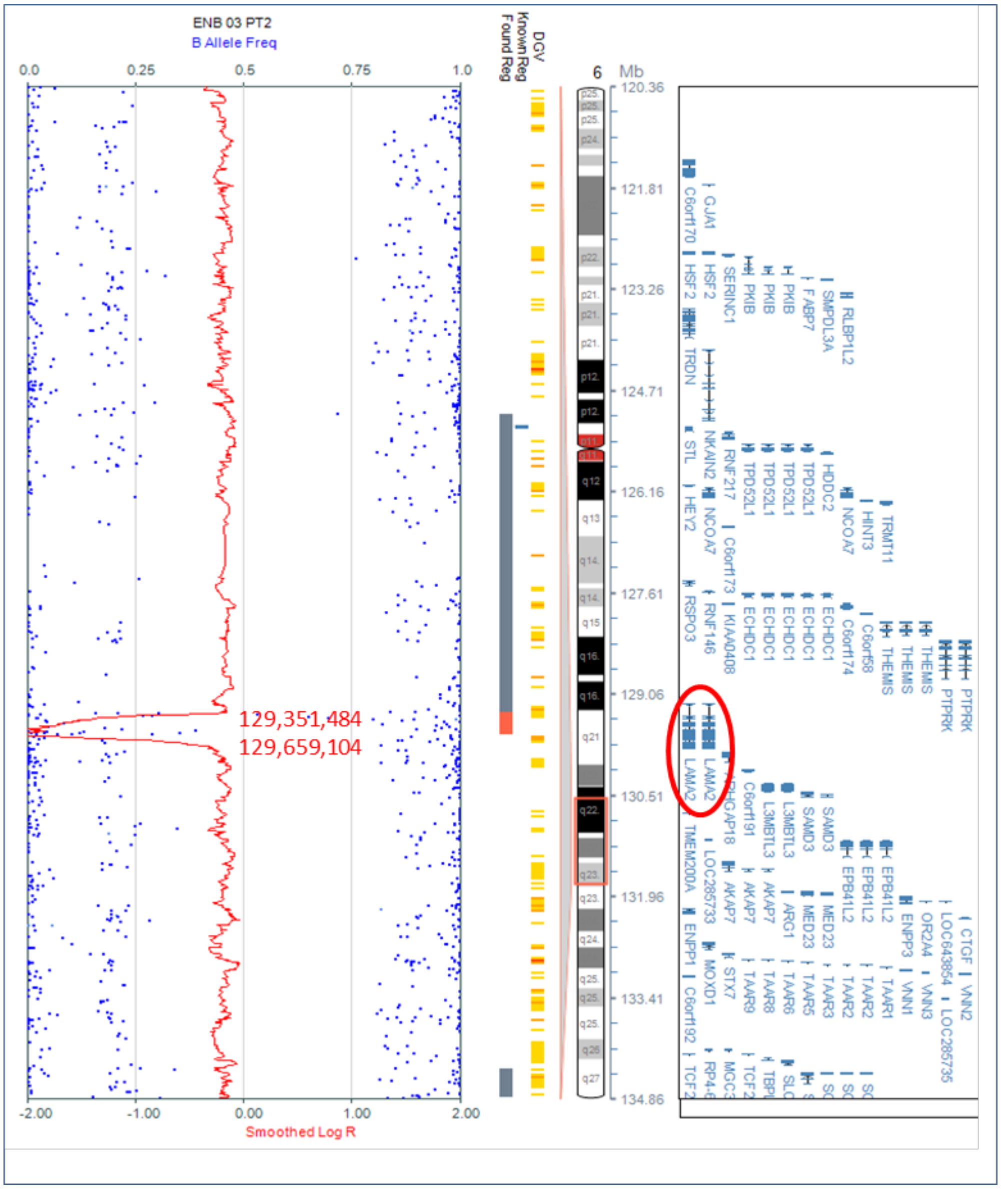

Supplementary Figure 2. Detection of a homozygous deletion in *LAMA2*. The panel on the left shows the array profile focusing on 6q22-23. The deletion is within the *LAMA2* gene, from genomic position 129,351,484-129,659,104. The position of *LAMA2* is chr 6: 129,204,286-129,837,710 (hg19).

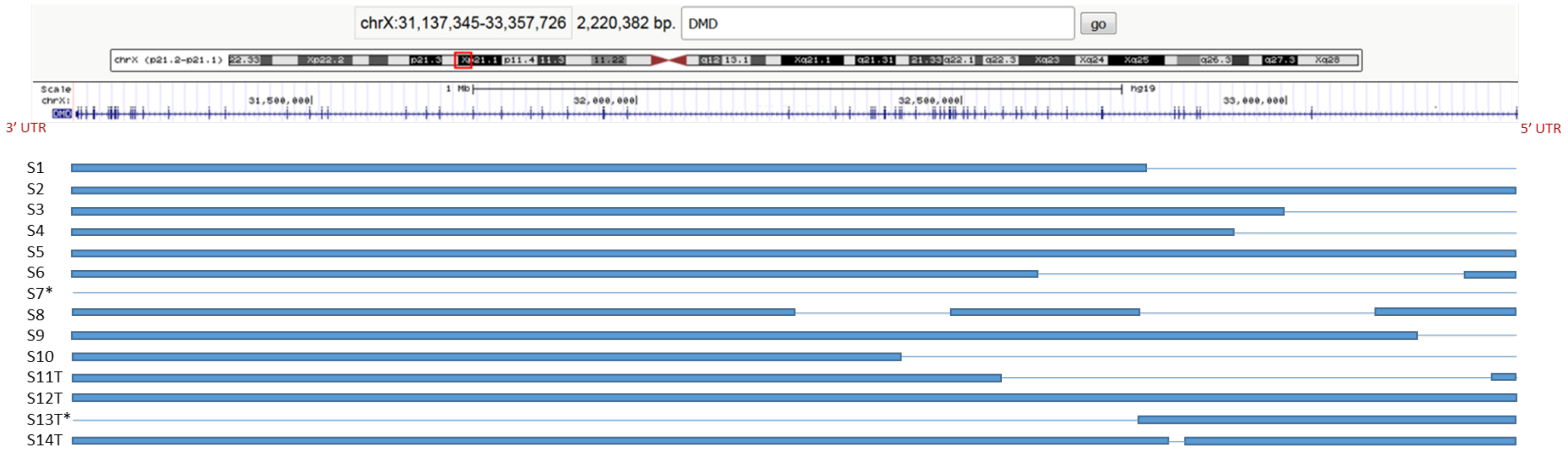

Supplementary Figure 3. Schematic summary of deletions in the *DMD* gene in 14 ONBs. Chromosome X and genome coordinates for *DMD* are shown on the top. *DMD* transcription is from the reverse strand, with 5' and 3' UTRs indicated for orientation. The thin lines represent *DMD* deletion in patient samples. Some deletions extend outside of the *DMD* gene (see **Table 1**). The deletion in sample 5 is 102 kb upstream to the *DMD* gene. Samples 7 and 13T were from female patients, with relative loss of chromosome X and the *DMD* gene, respectively.

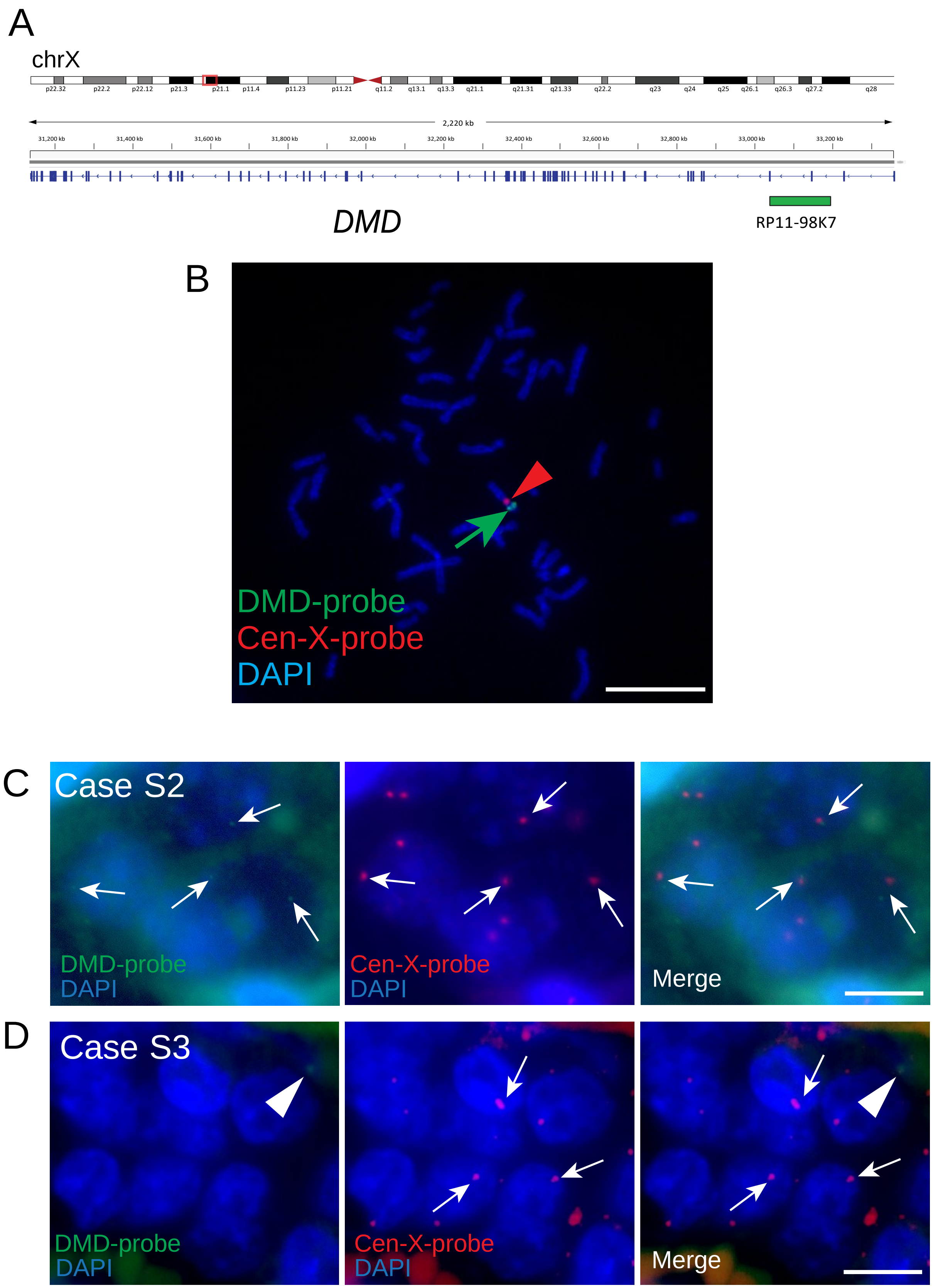
A
chrX
p22.32
p22.2
p22.12
p21.3
p21.1
p11.4
p11.23
p11.21
q11.2
q13.1
q13.3
q21.1
q21.31
q21.33
q22.2
q23
q24
q25
q26.1
q26.3
q27.2
q28
2,220 kb
31,200 kb
31,400 kb
31,600 kb
31,800 kb
32,000 kb
32,200 kb
32,400 kb
32,600 kb
32,800 kb
33,000 kb
33,200 kb
DMD
RP11-98K7
B
DMD-probe
Cen-X-probe
DAPI
C
Case S2
DMD-probe
DAPI
Cen-X-probe
DAPI
Merge
D
Case S3
DMD-probe
DAPI
Cen-X-probe
DAPI
Merge

Supplementary Figure 4. DMD locus-specific FISH. (A) Map indicating the location of the *DMD* locus-specific FISH probe used. (B) Correct Cen and *DMD* locus probe targeting to the X chromosome using normal human metaphase chromosomes. (C) Representative FISH image of a case in which no genetic deletions were detected at the *DMD* locus. (D) Representative FISH image of a case in which genetic deletion within the *DMD* locus was identified [leftmost panel; absence of green signals in tumor cells, presence of signal in adjacent benign stromal cell (arrowhead)]. In all cases, green punctate signals represent the *DMD* locus-specific probe, while red signals represent probe for the X centromere, which is included as an internal positive hybridization and signal proximity control. Scale bars = 10 μm.

Supplementary Table 1. Patient Characteristics

Specimen	Age (years)	Gender	Presentation	Prior Therapy	Stage†	Grade‡
ENB01PT	44	M	Recurrent	Surgery, Radiation	C	3
ENB03PT2	56	F	New	None	C	2
ENB04PT	56	M	New	None	D	3
ENB05PT	55	M	New	None	C	1
ENB7PT2	58	M	Recurrent	Surgery	C	3
ENB08PT2	51	M	New	None	C	2
ENB09PT1	40	F	New	None	A	2
ENB10PT	40	M	New	None	D	1
ENB11PT	33	M	New	None	C	2
ENB12PT	42	M	New	None	C	3
ENB606PT2	40	M	New	None	C	2
ENB1328	45	M	New	None	C	3
ENB1506	69	M	New	None	C	2
ENB2012-013	36	F	New	None	C	2
ENBBG	47	M	Recurrent	Radiation	C	2

†Modified Kadish staging: Stage A – tumor confined to the nasal cavity; Stage B – tumor confined to the nasal cavity and paranasal sinuses; Stage C – tumor extent beyond nasal cavity and paranasal sinuses, including involvement of the cribriform plate, base of the skull, orbit, or intracranial cavity; Stage D – tumor with metastasis to cervical lymph nodes or distant sites[1].

‡Histopathological grading was according to Hyams[2].

Supplementary Table 2. Summary of whole-exome sequencing analysis

WES Coverage Summary (eleven cases)	Tumor	Normal
Bases sequenced (after quality filtering)	$1.42X\ 10^{10}$	$1.50X\ 10^{10}$
Average # of reads per targeted base	140.0 ± 23.1	157.6 ± 18.9
Targeted bases with at least 10 reads (%)	91%	91%

WES Tumor and normal comparison	
Known SNPs identified in tumor	14,786 ± 682
Tumor SNPs identified in matched normal	14,775 ± 681
Non-synonymous somatic mutations in tumor	13.2 ± 5.6

Supplementary Table 3. Summary of whole-genome sequencing analysis

WGS Coverage Summary (six cases)	Tumor	Normal
Bases sequenced (after quality filtering)	1.25X 10^{11}	1.42X 10^{11}
Average # of reads per targeted base	29.0 ± 5.3	35.0 ± 4.6
Targeted bases with at least 10 reads (%)	93%	94%

WGS Tumor and normal comparison	
Known SNPs identified in tumor	45,649 ± 7452
Tumor SNPs identified in matched normal	45,649 ± 7452
Non-synonymous somatic mutations in tumor	15.5 ± 5.9

Supplementary References

1. Morita, A. *et al.* Esthesioneuroblastoma: prognosis and management. *Neurosurgery* **32**, 706-714 (1993).
2. Hyams, V. J. in *Special Tumors of the Head and Neck* (eds J.G. Batsakis, V.J. Hyams, & A.R. Morales) 24-29 (ASCP Press, 1983).
