# Supplementary material for: Genomic analysis identifies frequent deletions of Dystrophin in olfactory neuroblastoma
Source: Nat Commun. 2018 Dec 21;9:5410. doi: 10.1038/s41467-018-07578-z (PMC6303314; doi:10.1038/s41467-018-07578-z)
Supplement: Supplementary file 3 — Description of Additional Supplementary Files [file 41467_2018_7578_MOESM3_ESM.docx]

Description of Additional Files

**Dataset 1:** Somatic Mutations Detected Via Whole-Exome Sequencing
